# Long-Term Outcomes and Cost-Effectiveness of an Internet-Based Self-Help Intervention for Social Anxiety Disorder in University Students: Results of a Randomized Controlled Trial

**DOI:** 10.1155/2023/7912017

**Published:** 2023-11-17

**Authors:** Fanny Kählke, Claudia Buntrock, Filip Smit, Thomas Berger, Harald Baumeister, David Daniel Ebert

**Affiliations:** ^1^School of Medicine and Health, Department Health and Sport Sciences, Professorship for Psychology and Digital Mental Health Care, Technical University Munich, Munich, Germany; ^2^Institute for Social Medicine and Health Systems Research, Otto-von-Guericke-Universität Magdeburg, Magdeburg, Germany; ^3^Department of Clinical, Neuro and Developmental Psychology, Amsterdam Public Health Research Institute, Vrije Universiteit, Amsterdam, Netherlands; ^4^Department of Clinical Psychology and Psychotherapy, Universität Bern, Bern, Switzerland; ^5^Department of Clinical Psychology and Psychotherapy, Ulm University, Ulm, Germany

## Abstract

Social anxiety disorder (SAD) is widespread among university students and is associated with high costs for the society. While unguided internet- and mobile-based interventions (IMIs) may have short-term effects in reducing SAD symptoms, evidence for their long-term efficacy and cost-effectiveness is still limited. The aim of this study is to examine the 6-month outcomes of an IMI for university students with SAD. Participants were recruited via mass mails sent to enrolled students and included if they were at least 18 years old, met the diagnostic criteria of SAD in a structured clinical interview for DSM-IV axis I disorders (SCID-I), and provided written informed consent. In a prospective study designed as a two-armed randomized-controlled trial, 200 students (mean age 26.7 years) diagnosed with SAD were randomly assigned to an IMI or a waitlist control (WLC) condition. The IMI consisted of nine weekly sessions based on the cognitive-behavioral treatment model for social phobia by Clark and Wells. The primary outcome was SAD symptom severity assessed via the Social Phobia Scale (SPS) and the Social Interaction Anxiety Scale (SIAS). A health economic evaluation from a societal and healthcare perspective examined the costs related to the symptom-free status and quality-adjusted life years (QALYs) gained. Statistically significant differences in SAD symptom severity previously found at posttreatment favoring the IMI were maintained at a 6-month follow-up [SIAS (Cohen's *d* = 0.59; 95% CI, 0.30, 0.87) and SPS (Cohen's *d* = 0.83; 95% CI, 0.54, 1.1)]. From a societal perspective, at a willingness to pay (WTP) of €0, the intervention was found to have a 92% and 93% probability of cost-effectiveness compared with the WLC per symptom-free status and QALY gained, respectively. From a healthcare perspective, the likelihood of cost-effectiveness of the intervention was 97% per symptom-free status at a WTP of €1000 (US$1326) and 96% per QALY gained at a WTP of €6000 (US$7956). This IMI is effective in treating university students with SAD and has an acceptable likelihood of cost-effectiveness compared with WLC from a societal perspective. This intervention can be integrated into university healthcare to reach students with SAD as it is scalable, shows a high probability of cost-effectiveness, and overcomes known treatment barriers. This trial is registered with DRKS00011424.

## 1. Introduction

Social anxiety disorder (SAD) is a prevalent and impairing disorder and is considered a public health concern. In particular, university students fall within the age range when common mental health problems reach their developmental peak [1]. Being at a university is associated with many stressors and transitional events [2]. In Germany, persons aged 18–35 show a 4.8% [3] 12-month prevalence of SAD, while 12.2% of university students show clinically relevant social phobic symptoms [4]. Several adverse effects on quality of life (QoL) and identity formation [5], alcohol consumption [6], and suicidal ideation [7] are reported in university students with SAD. These may lead to premature dropout and academic underachievement [8]. Moreover, SAD is associated with substantial impairment across several domains, such as relationship, daily and social life, and work [9]. Altogether, it generates direct (treatment), indirect (productivity losses, absence of work, and low qualification [10, 11]), and intangible costs (lower QoL and social impairment). In Germany, the mean total 6-month costs for SAD in a clinical sample were estimated at €4802 per person, which are mainly attributed to indirect cost [12].

While the university setting enables comprehensive approaches for prevention, early intervention, and treatment of students with SAD, students in general are averse to face-to-face counseling centers preferring to solve their problems themselves [13]. In particular, students with SAD face attitudinal barriers (fear of stigmatization and negative evaluation) to help-seeking [14] that may leave them untreated with a chronic condition [15].

Internet- and mobile-based interventions (IMIs), which are flexible, accessible, and anonymous [16–18], present a promising approach to reach those affected individuals. Unguided IMIs have received adequate attention owing to their potential for high scalability and relatively low marginal costs. The efficacy of unguided IMIs based on cognitive-behavioral approaches targeting SAD has been shown with medium effects at posttreatment compared with that of passive controls (*g* = 0.78, 95% CI [0.50–1.05], SE = 0.14, *p* < 0.001, *k* = 5). However, in contrast to guided IMIs, the evidence for the long-term efficacy of unguided IMIs is still limited [19–22].

Moreover, the value of IMIs for SAD in university students has not been sufficiently investigated. Existing evidence suggests that IMIs targeting SAD in university students might have beneficial effects up to one year with or without guidance [23–25]. However, the evidence base is weak because studies mainly focused on fear of public speaking [23, 25], had substantial dropout rates [24, 25], and were underpowered [23].

Apart from the efficacy of IMIs for SAD, evidence to support their cost-effectiveness is still insufficient. While the assessment of the efficacy takes the benefits for patients into account, the assessment of economic consequences also considers a wider perspective by providing insights into societal costs and benefits. Only three studies investigated the economic merits of IMIs for SAD, indicating that guided [26–28] and unguided [29] IMIs may be a cost-effective treatment option in the long term (≥6 months) in the general population. However, it should be considered that evidence is still limited due to high dropout rates [29], few statistical analyses, and the absence of a full health economic evaluation [28].

Previously published results of this study (10 weeks after randomization) showed that IMI was superior to a waitlist control (WLC) group in reducing SAD symptom severity in university students with moderate to large effect sizes [30]. In this study, we report its clinical effects at 6-month follow-up and the health economic outcomes from a societal and public healthcare perspective over a 6-month period.

## 2. Methods

### 2.1. Study Design and Participants

**Figure 1:**
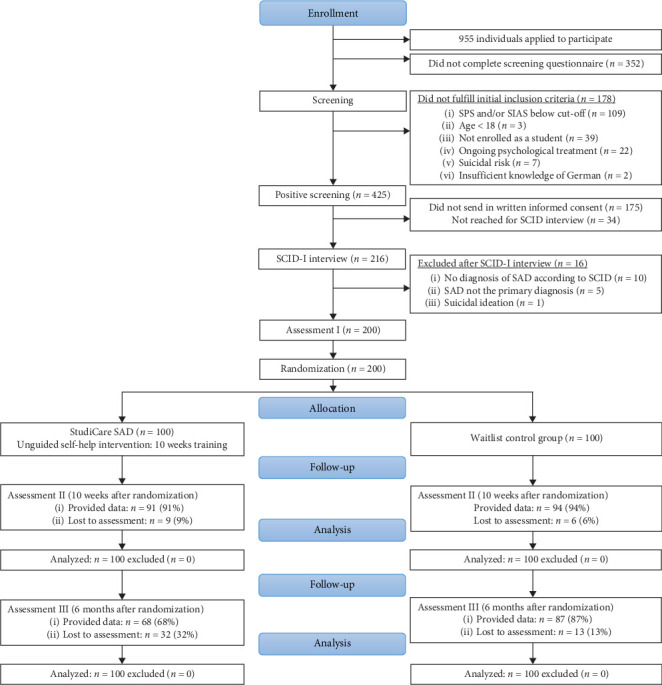
Flow of participants.

A two-arm randomized controlled design was used to allocate 200 participants (block size of 8, varying ratio) to either the intervention (IMI) or control (WLC) group [31]. Alongside this trial, a cost-effectiveness analysis was conducted. An independent investigator randomized participants applying the RandList program [32]. Self-reported measurements were collected over three measurement points using a secured web-based assessment system (UNIPARK [33], 256-bit encrypted): at baseline (T0), posttreatment (T1; 10 weeks after randomization), and 6-month follow-up (T2) (Figure 1). The participants were recruited via e-mails sent to enrolled students at universities in Switzerland (*N* = 3), Austria (*N* = 2), and Germany (*N* = 8) from January 2017 to February 2018. The applicants met the following inclusion criteria: they were 18 years or older, scored >21 on the Social Phobia Scale (SPS) and/or>32 on the Social Interaction Anxiety Scale (SIAS), met the diagnostic criteria of SAD according to the structured clinical interview for DSM-IV axis I disorders (SCID-I), and provided written informed consent. The interviews were conducted by trained interviewers via telephone [34]. The exclusion criteria included individuals at risk of suicide, showing dissociative symptoms, being diagnosed with psychosis, or currently undergoing psychotherapy. The Ethics Committee of the Friedrich-Alexander-University Erlangen-Nuremberg, Germany, approved the study. The trial was registered in the German clinical trial registry (DRKS00011424) on December 14^th^, 2016. More details on the study design, diagnostic interview, and participants can be found in the study protocol [31].

### 2.2. Intervention

The intervention consisted of nine weekly sessions (approx. 60 minutes each) based on the cognitive-behavioral treatment of Clark and Wells [35, 36]. It contained text-based information material, various exercises (e.g., attention training), and diaries (e.g., identifying and questioning negative thoughts). The sessions were based on motivational enhancement (Session 1); psychoeducation (Session 2); identification and modification of negative thoughts through a thought diary (Sessions 3) and cognitions related to fear of positive evaluation (FPE) (Session 4); exercises to reduce self-focused attention, including behavioral experiments, such as in vivo exposures (Sessions 5–7); healthy lifestyle and problem-solving skills (Session 8); and relapse prevention (Session 9). The focus on FPE reflected a new treatment element that included the core components of SAD: fear of negative and positive evaluation [37]. A detailed description of the intervention can be found elsewhere [31].

## 3. Outcome Measures

### 3.1. Primary Outcome

The level of social anxiety was measured by the SPS and the SIAS [38]. These two self-report questionnaires complement each other and are usually administered together. The SIAS assesses more general fears of social interaction, whereas the SPS focuses on fears of being judged by others during daily activities. Each scale consists of 20 items rated on a 5-point Likert scale (0 = “not at all” to 4 = “extremely”). These two scales have been found to be valid, reliable, and useful for clinical and research purposes [39]. Cronbach's *α* for the SIAS and SPS ranged from 0.90 to 0.94 [40] (current sample, *α* = 0.91–0.93). Symptom-free status was operationalized as scoring ≤ 17 on the SPS and ≤26 on the SIAS [41]. Stangier et al. [38] proposed these cut-off values to differentiate between social phobic cases and non-social phobic cases. Diagnostic status was assessed via a diagnostic interview (SCID-I) by an interviewer blinded to the treatment condition after 6 months. Interrater reliability was evaluated in 20% of randomly selected participants.

### 3.2. Secondary Outcomes

The secondary outcomes are as follows:

- Symptoms of social anxiety (the Liebowitz Social Anxiety Scale [LSAS-SR] assesses fear and avoidance in 24 different situations) [42] (Cronbach's *α* = 0.95 in the current sample)
- Fear of positive evaluation (Fear of Positive Evaluation Scale [FPES] showing good psychometric properties in clinical and healthy samples [10 items] [43, 44] [*α* = 0.79], Disqualification of Positive Social Outcomes Scale [DPSOS] [13 items] [45] [*α* = 0.90])
- Depressive symptoms (Beck Depression Inventory II [BDI-II] [21 items] has shown high reliability and validity in SAD clients) [36, 46] (*α* = 0.91)
- General psychopathology (Brief Symptom Inventory [BSI] [53 items, 9 dimensions] and Global Severity Index [GSI] as overall mean score reported and robust psychometric properties) [47, 48] (*α* = 0.96)
- Interpersonal problems (Inventory of Interpersonal Problems [IIP-64] [8 dimensions] has shown adequate psychometric properties) [49, 50] (*α* = 0.96)

## 4. Health Economic Evaluation

### 4.1. Quality-Adjusted Life Years (QALYs)

QALYs were computed using the Assessment of Quality of Life (AQoL-8D) and the EuroQol (EQ-5D-5L) instruments. The AQoL-8D assesses eight dimensions (independent living, pain, senses, mental health, happiness, coping, relationships, and self-worth) and is a reliable and valid instrument [51] with Cronbach's *α* of 0.96. The EQ-5D-5L is a widely applied, valid, and reliable measurement of QoL [52]. It consists of five items on a 5-point Likert scale related to mobility, self-care, common activities, pain/discomfort, and anxiety/depression. Utility values were derived using instrument-specific utility weights (EQ-5D [53] and AQoL [54]). QALYs were calculated using the area-under-the-curve (AUC) method of linearly interpolated utilities between measurements to cover the whole 6-month follow-up period. The EQ-5D was only used for sensitivity analyses.

### 4.2. Costs

We retrospectively assessed the 3-month healthcare costs, productivity losses, and patient and family costs using the “Trimbos Institute and Institute of Medical Technology Questionnaire for Costs Associated with Psychiatric Illness” (TiC-P) adapted to the German healthcare system [55]. The TiC-P is a frequently used and reliable self-report instrument for healthcare utilization and productivity losses among (German) [56, 57] patients with mental illnesses [58]. For each participant, the units of resource use were multiplied by the standard unit cost prices [59]. The intervention's market price was estimated at €150 (US$198.89) per participant, reflecting costs due to maintenance and hosting. In addition to the German value added tax of 19%, the interventions costs were €178.50 (US$236.68). It was presumed that each participant had a computer and internet access. The costs of therapeutic appliances and medication were obtained from the Lauer-Taxe [60] and calculated according to the method of Bock et al. [59]. German healthcare costs were last published in 2011 and therefore indexed from 2011 to 2017 (index factor of 1.09) according to the German consumer price index [61].

Costs stemming from productivity losses through absenteeism and presenteeism were only assessed in students who had a paid job. Using the human capital approach [62], absenteeism costs were calculated as the number of working days lost due to illness multiplied by the average gross daily wage of the student's monthly salary. Presenteeism was determined based on an inefficiency score (Osterhaus method [63]) multiplied by the number of working days affected. Then, costs of presenteeism were calculated based on the student's gross daily wages. Productivity losses generated by unpaid work, such as daily chores, were valued using a shadow price of €19.63 (US$26.03) per hour for domestic help. Table S1 shows additional costing information.

The AUC method was applied to estimate cumulated costs by linearly interpolating costs over a 3-month period measured at baseline, post, and follow-up to cover the entire 6-month follow-up period [64]. All costs were expressed in Euros (€) for 2017 (December), the year in which the study was conducted. Purchasing power parities based on the Organization for Economic Cooperation and Development were used to convert costs to US dollars [65] (reference year: 2017; €1 was equated to US$1.33). The resource utilization in Austria and Switzerland was valued using German standard unit cost prices to increase consistency in costs and minimize confounding.

### 4.3. Evaluation of Clinical Outcomes

This study was conducted to detect a mean standardized difference of *d* = 0.40 in the primary outcomes (SPS/SIAS) between the groups at post-measurement [31]. The results were reported according to the Consolidated Standards of Reporting Trials statement [66] using intention-to-treat (ITT) procedures. Missing clinical outcome data were imputed by applying a Markov Chain Monte Carlo multivariate imputation algorithm with 10 estimations per missing value [67].

The evaluation of the clinical outcomes was performed using the SPSS software [68]. The IMI and WLC were compared six months after randomization (T2) using analysis of covariance (ANCOVA) with baseline levels as covariates. Due to the violation of normally distributed error terms of many outcomes, robust ANCOVA was used [69]. These analyses were adjusted for multiple testing. Hence, *α* was set at a level of <0.025 [70] for testing the primary outcomes and <0.05 for all other tests. Cohen's *d* with 95% CIs was calculated.

Treatment response and clinically significant deterioration were defined by the Reliable Change Index [38]. The participants were defined as reliably improved if their SPS (SIAS) score declined from baseline to 6-month follow-up with more than 1.96 standard units, while also considering the reliability of the measurement instruments to compensate for random measurement error. The participants met the criteria for reliable change when they had improved (deteriorated) at least 7.03 points on the SPS and 9.53 points on the SIAS. Moreover, the participants were rated as symptom-free if they scored 17 or below on the SPS and 26 or below on the SIAS [38]. To further guide the clinical interpretation, the numbers needed to treat (NNT) were calculated [71, 72]. Differences in symptom-free status, reliable change, and diagnostic status as assessed by SCID interviews between the groups were assessed at follow-up using the chi-squared test.

### 4.4. Health Economic Evaluation

The evaluation adhered to standards set by the Consolidated Health Economic Evaluation Reporting Standard (CHEERS) and International Society for Pharmacoeconomics and Outcomes Research (ISPOR RCT-CEA Task Force Report) [73, 74].

All data was analyzed based on the ITT principle. Thus, missing cost data was imputed using the regression imputation procedure using the predictors of the outcome (e.g., baseline costs, annual gross salary, and status of employment) and dropout rate (e.g., sex and age) that were identified via logistic regression analysis.

The ordinary least square (OLS) regression was used to estimate QALYs while controlling for baseline utility values. From a societal and public healthcare perspective, cost categories and cost per study arm were evaluated by OLS regression models. One cost outlier was identified by calculating the Mahalanobis distance based on the total cost (*N* = 1, in the WLC) and handled using winsorization where it was substituted by the value at the 99th percentile [75]. No discounting of costs and effects was applied because the follow-up period did not exceed one year. The incremental cost-effectiveness ratio (ICER) displays the incremental costs per unit of the effect (QALY, symptom-free status). The ICER was calculated as ICER = (costs_IMI_ − costs_WLC_)/(effects_IMI_ − effects_WLC_), where the costs are cumulated over a period of 6 months and effects are reflected by the symptom-free status or QALY gains.

For our economic analyses, we applied a probabilistic decision-making approach [76] that accounts for stochastic uncertainty [77] in the study data. It provides the decision-maker with information about probabilities rather than statistical significance. A 5000-fold bootstrapped seemingly unrelated regression equation model on costs and effects was used to generate the incremental costs and effects, while adjusting for baseline utilities, age, and prior psychotherapy [78]. The 5000 bootstrap replications of cost-and-effect pairs were used to obtain 95% confidence intervals and plotted in a cost-effectiveness plane. The plane depicts the incremental effects between the intervention and control group on the *x*-axis and the incremental costs between the groups on the *y*-axis. The intervention “dominates” the control groups if better effects are obtained for lower costs. Hence, the majority of simulated ICER falls in the southeast quadrant. In contrast, in the northwest quadrant, the intervention is “inferior” to the control group as higher costs are associated with worse health outcomes. Thus, it is not considered cost-effective [62]. In the southwest quadrant, an intervention is less effective and less costly than the control group. On the other hand, in the northeast quadrant, an intervention is more effective and more costly than the control condition. Here, the amount of money a decision-maker is willing to pay for one additional positive outcome determines the adoption of a new intervention. A cost-effectiveness acceptability curve was displayed that indicates the probability of cost-effectiveness of an IMI at varying WTP ceilings, given that there is no common threshold for gaining one unit of health (e.g., symptom-free status). Analyses were carried out with Stata version 16.1 [79].

### 4.5. Sensitivity Analyses

The sensitivity analyses were conducted to inspect the robustness of our results. First, the data of the participants who completed the 6-month follow-up assessment were analyzed. Second, changing market prices can lead to varying intervention costs, which explains why the increased intervention costs were examined (+50%, 100%). Third, Swiss students (*n* = 40, 20%) were excluded to validate the robustness of the findings. Their number and employment rate were balanced across groups, but differences in healthcare settings and salaries could have biased the results. Fourth, to facilitate comparability across studies, the widely applied EQ-5D instrument was used to generate QALYs. Lastly, the diagnostic status as a meaningful effect outcome for policymakers was used for the cost-effectiveness analysis.

## 5. Results

### 5.1. Sample

The sample predominately consisted of female (*n* = 124, 62%) German university students (*n* = 150, 75%) aged 27 (SD 6.34) (Table S2). A comprehensive description of the study sample and the participant flow can be found elsewhere [31]. We did not observe any clinically relevant baseline differences between the study conditions. The dropout rates between IMI (*n* = 32/100, 32%) and WLC (*n* = 13/100, 13%) differed significantly (*χ*^2^ = 10.35; df = 1; *p* < 0.01), yet the dropout rate was not associated with the sociodemographic factors or the baseline SAD symptoms. The attrition rate was 22.5% (45/200) at the 6-month follow-up.

### 5.2. Outcome Measures

As shown in Table 1, the IMI was associated with lower scores on both primary outcome measures than WLC. These between-group differences were statistically significant: SPS, *F*(1, 197) = 55.01, *p* < 0.001; SIAS, *F*(1, 197) = 49.03, *p* < 0.001. The corresponding standardized effect sizes were moderate for SIAS (*d* = 0.59, 95% CI [0.30, 0.87]) and large for SPS (*d* = 0.83, 95% CI [0.54, 1.10]). Fewer participants in the IMI (*n* = 30/100) than in the WLC (*n* = 60/100) presented with a clinical diagnosis of social phobia assessed through a SCID interview (*χ*^2^ = 18.18; df = 1; *p* < 0.001). The inter-rater reliability showed substantial agreement (Cohen's kappa, *κ* = 0.78 [80]).

### 5.3. Treatment Response, Symptom-Free Status, and Symptom Deterioration

After 6 months, significantly more participants in the IMI showed a reliable improvement and achieved a symptom-free status compared with those in the WLC based on the SPS and the SIAS. Likewise, the clinically significant deterioration was lower in the IMI compared with the WLC for both outcomes (Table S3).

### 5.4. Secondary Outcome Analyses

Table 2 shows the results for the secondary outcomes, interpersonal problems, depression, somatic symptoms, FPE, and QoL. Significant between-group differences for all outcomes, except the EQ-5D, with effect sizes ranging from *d* = 0.23 (95% CI [0.05, 0.50]) for the AQoL to *d* = 0.76 (95% CI [0.47, 1.05]) for the LSAS-SR were observed.

## 6. Health Economic Evaluation

### 6.1. Health Outcomes

Regarding symptom-free status, the IMI significantly differed from WLC on the SPS [incremental effect (Δ[E] = 0.26; 95% CI, 0.15–0.37)] and on the SIAS [incremental effect (Δ[E] = 0.24; 95% CI, 0.14–0.34)]. On average, the participants in the IMI gained 0.66 QALYs (95% CI, 0.64–0.67) during follow-up, whereas the participants in the WLC gained 0.61 QALYs (95% CI, 0.59–0.62). Statistically significant differences in the adjusted incremental QALYs were observed (Δ[E] = 0.046; 95% CI, 0.02–0.07).

### 6.2. Costs

At baseline, the mean total costs only differed (€138; US$183) moderately between the IMI (€464, US$615) and the WLC (€603; US$800).  Table 3 displays the average accumulated costs over a 6-month period per participant by study arm. After 6 months, the total incremental costs were -€211 in favor of the intervention group (IMI, €850; WLC, €1061). The average healthcare costs were higher in the IMI (€345) compared with the WLC (€240). The patient and family costs were similar in both groups slightly favoring the IMI. Productivity losses especially presenteeism at work produced the highest cost differences of -€227 (IMI, €391; WLC, €618) exceeding the intervention costs.

### 6.3. Societal Perspective

**Figure 2:**
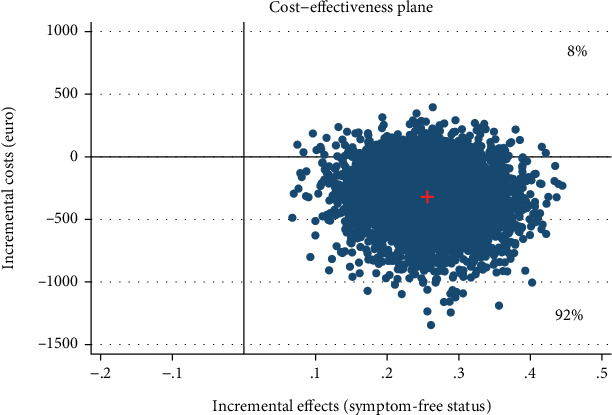
Scatter plot showing the mean differences in costs and effect outcome (symptom-free status, SPS) data using 5000 bootstrap replications from a societal perspective.

**Figure 3:**
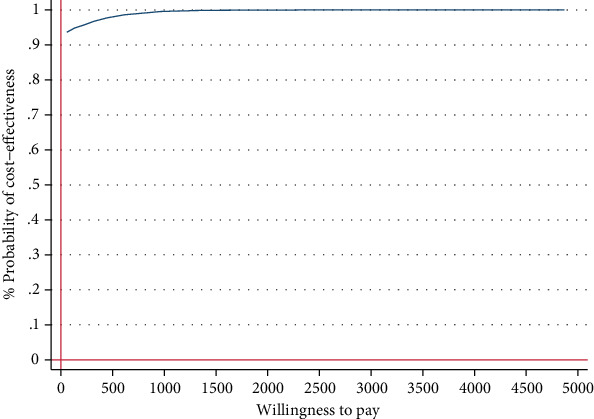
Cost-effectiveness acceptability curve showing the probability of the IMI being cost-effective at varying WTP ceilings (based on 5000 replicates of the ICER using mean differences in costs and symptom-free status based on SPS) from a societal perspective.

Table 4 shows the incremental costs, effects, and ICERs based on the 5000 bootstraps. The IMI dominated the WLC related to the symptom-free status with larger effects on the SPS and SIAS and less costs (SPS, -€321, 95% CI [−862, 66]; SIAS, -€324, 95% CI [−774, 125]). In the cost-effectiveness plane, the majority of ICERs fell under the southeast quadrant (Figure 2), reflecting a 92% probability that the intervention generates greater health effects at lower costs than WLC (Figure 3).

**Figure 4:**
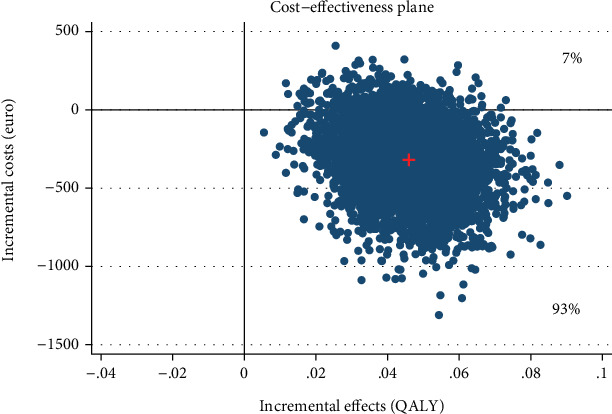
Scatter plot showing the mean differences in costs and effect outcome (AQoL QALY) data using 5000 bootstrap replications from a societal perspective.

**Figure 5:**
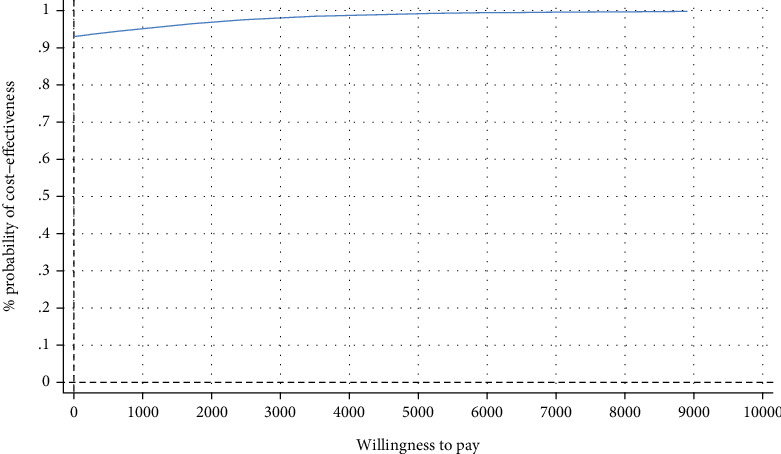
Cost-effectiveness acceptability curve showing the probability of the IMI being cost-effective at varying WTP ceilings [based on 5000 replicates of the incremental cost-effectiveness ratio (ICER) using mean differences in costs and QALYs] from a societal perspective.

The IMI generated small QALY gains at lower costs (-€319, 95% CI, −831–64) compared with the WLC. From a societal perspective, 93% of the simulated ICERs fell under the southeast quadrant reflecting the intervention's probability of dominating WLC (Figure 4). Assuming a WTP of €1000 for QALY gains, the probability rose to 95% (Figure 5).

### 6.4. Healthcare Perspective

**Figure 6:**
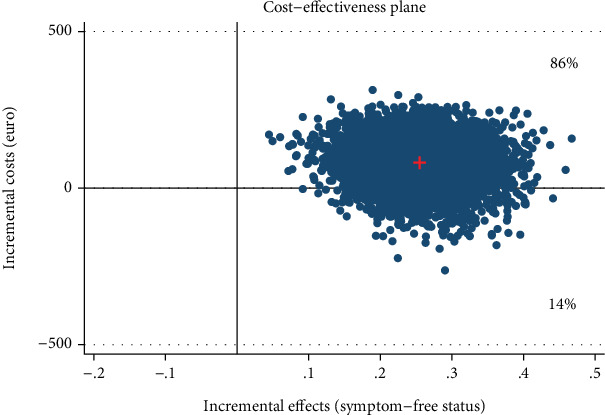
Scatter plot showing the mean differences in costs and effect outcome (symptom-free status, SPS) data using 5000 bootstrap replications from a healthcare perspective.

**Figure 7:**
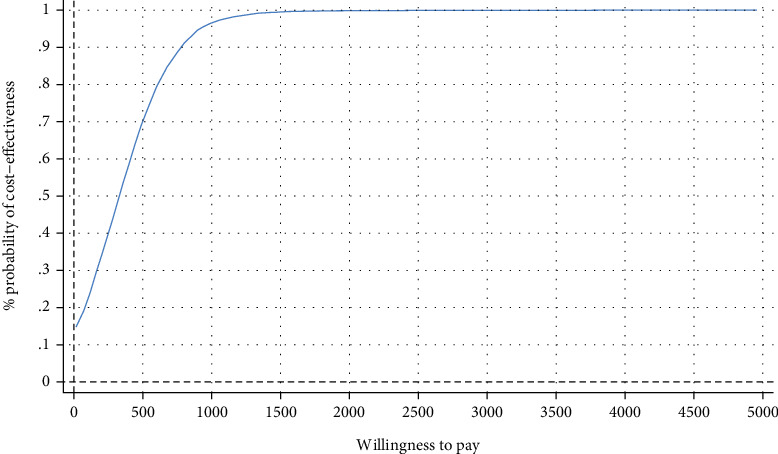
Cost-effectiveness acceptability curve showing the probability of the IMI being cost-effective at varying WTP ceilings (based on 5000 replicates of the ICER using mean differences in costs and symptom-free status based on SPS) from a healthcare perspective.

**Figure 8:**
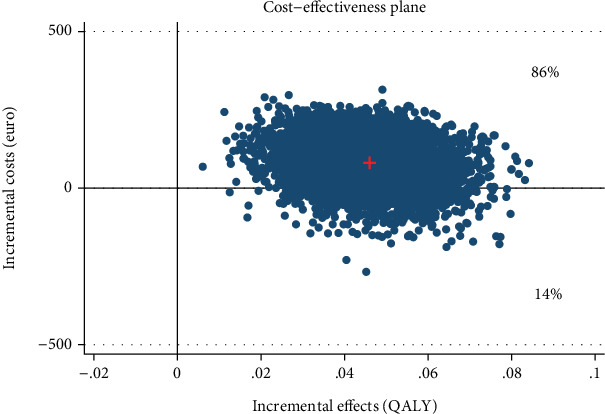
Scatter plot showing the mean differences in costs and effect outcome (AQoL QALY) data using 5000 bootstrap replications from a healthcare perspective.

**Figure 9:**
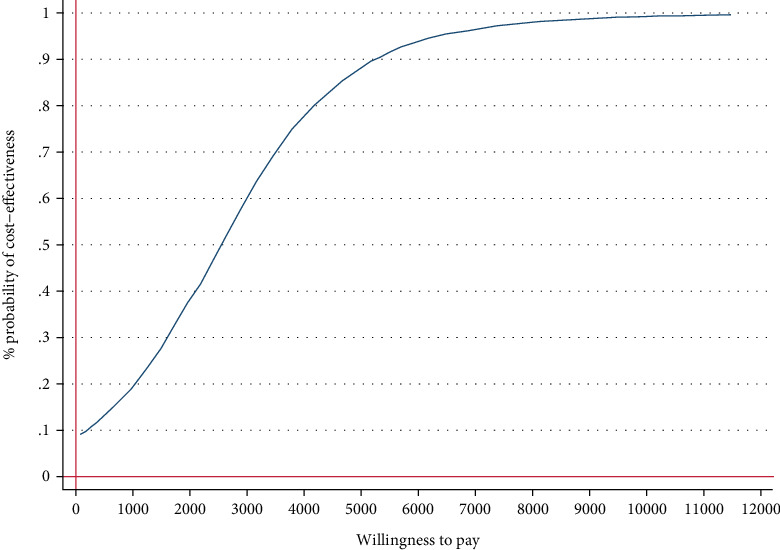
Cost-effectiveness acceptability curve showing the probability of the IMI being cost-effective at varying WTP ceilings [based on 5000 replicates of the incremental cost-effectiveness ratio (ICER) using mean differences in costs and QALYs] from a healthcare perspective.

The bootstrapped ICER related to the symptom-free status on the SPS and SIAS yielded an ICER of €348 (95% CI, SPS [−284,1069]; SIAS [304, 1043]), indicating that the IMI generated larger effects at higher costs (SPS, €81; SIAS, €79) compared with the WLC. Hence, the majority of ICERs fell under the northeast quadrant (86%), while the probability of cost-effectiveness of the intervention compared to WLC rose from 70/69% at a WTP of €500 to 97% at a WTP of €1000 (Figures 6 and 7). Regarding the cost utility, the IMI generated higher effects per QALY gained at higher costs compared with WLC (€81; 95% CI, 105–200). Thus, 86% of the simulated ICERs fell in the northeast quadrant, showing higher QALY gains and costs (Figure 8). At a WTP of €0, €2000, and €6000 for gaining one QALY, the probability rose from 14% to 54% to 96% (Figure 9).

### 6.5. Sensitivity Analyses

First, the study completers generated significant effects on all assessed outcomes and effect sizes at least as large as in the ITT analysis (±*d* = 0.1). Second, regarding the QALY gains, even increasing the invention costs by 50% and 100% did not alter the interpretation of results either from a societal or healthcare perspective, respectively (Table 2). Third, excluding the Swiss students did not affect the results of the CEA or the CUA analyses. Fourth, using the EQ-5D-5L resulted in a slightly nonsignificant (*t*_200_ = −0.65, *p* = 0.52) incremental QALY gain of the IMI (0.939 QALY, SD 0.070) compared with the WLC (0.932 QALY, SD 0.865). The greater sensitivity of the AQoL instrument and the potential ceiling effect of the EQ-5D instrument may have led to the differences between the AQoL QALYs (>0.55) and the EQ-5D QALYs (>0.9). Nevertheless, for gaining a QALY at a WTP of €0/€10,000, the probability of cost-effectiveness was similar (99%/89%) from a societal perspective and lower (15%/26%) from a healthcare perspective compared with the AQoL QALYs. Fifth, using the diagnostic status for the health economic evaluation yielded similar results to the symptom-free status (Table 4).

## 7. Discussion

### 7.1. Principal Findings

This study is the first to evaluate the long-term efficacy and the cost-effectiveness of an unguided IMI for university students with SAD compared with a waitlist control condition (WLC) with unrestricted access to treatment as usual over 6 months from a societal and public healthcare perspective. The IMI maintained a significant and favorable effect on social phobia symptoms with moderate to large effect sizes between groups at follow-up assessment (6 months; SPS, *d* = 0.83; SIAS, *d* = 0.59) compared with the WLC. The IMI generated slightly lower costs (-€321; 95% CI, −862–66), more QALYs (0.046; 95% CI, 0.024–0.68), and symptom-free status (SPS = 0.26; 95% CI, 0.15–0.37) compared with the WLC in the long term. From a societal perspective, the IMI dominated the WLC, while from a healthcare perspective, the probability for cost-effectiveness was 96% at a WTP of €6000 (US$7956) per symptom-free status and QALY. Our findings were robust to sensitivity analyses.

### 7.2. Comparison with Prior Work

Our findings are consistent with the evidence on the efficacy of unguided IMIs in the general population suffering from SAD in the long term [19]. A study (*N* = 81) compared an unguided IMI with two types of guided self-help after 6 months [36] and found within-group effects (*d* ≈ 1.5) similar to our study, but no significant effect was observed between the groups. Likewise, smaller but also persistent effects (*d* = 0.2) of an unguided IMI were found when compared with WLC after one year [29].

Regarding the university students, our findings support the existing evidence for internet-based interventions targeting SAD showing similar results as the previous studies [24] (some focusing on fear of public speaking [23, 25]). Furthermore, these studies are characterized by a substantial dropout rate at posttreatment [24] (40%) or follow-up [25] (67%) and did not assess the long-term efficacy of the intervention.

Likewise, further research is needed to confirm the economic benefits of IMIs for SAD. Results from our trial add to the converging evidence pointing to their cost-effectiveness. Four studies reported on health economic outcomes of IMIs in SAD [26–29]. Three studies found that guided IMIs might be a cost-effective approach compared with an active control over a period of 6 months and 4 years from a societal and provider perspective, respectively. Showing similar results to our study, one guided IMI generated less costs and better treatment outcomes. At a WTP of 0 Euro, this IMI showed an 81% probability of cost-effectiveness at 6 months [25] and a 61% probability at a 4-year follow-up [27]. Another guided IMI compared with face-to-face treatment was judged as cost-effective, only including cost for therapist time [27].

To our knowledge, only one health economic evaluation has compared unguided IMI and passive control [29], which indicated that the intervention is likely to be cost-effective. The study differs from the present study in terms of the general population, higher average age, different inclusion criteria (also subclinical participants), and instruments used (SF-6D versus AQoL). The IMI generated higher costs and better effects at 6 months and dominated the control condition after 12 months. Compared with our study, this trial focused on a nonclinical sample and had a substantial dropout rate (50%) which could lead to selection bias by attrition.

### 7.3. Strengths and Limitations

This study has several strengths and limitations. The data in our trial was based on self-report measures and diagnostic interviews, thereby increasing the robustness of the results while minimizing bias accompanying self-reported data due to recall period or selective recall. Evidence-based assessment consisting of more than one assessment technique is recommended for accurate assessment and effective treatment of anxiety disorders [81]. Additionally, our study only showed a relatively small number of dropouts (22.5%) at 6-month follow-up compared with most unguided studies [82]. This could be due to our structured research process (e.g., diagnostic interviews) and the highly educated and technologically sophisticated group of participants. Moreover, the characteristics of the study groups were well balanced at baseline, and the proportion of female participants (62%) was relatively low compared with most IMIs also reflecting the distribution of SAD in the population. A further strength of our study is the inclusion of a full economic evaluation. Notably, the economic findings were consistent across various sensitivity analyses.

However, several limitations were noted. First, the time horizon of our study was limited to 6 months. Costs due to present and future underachievement, prolonged studies, and study dropouts resulting in lower academic qualifications could not be captured nor included in our evaluations and may even increase the impact of the intervention. Second, most instruments used self-report measures, which might have led to “social desirability” and “recall bias.” Third, there are several possible reasons why our study generated relatively high effect sizes compared to other IMIs targeting SAD. Significant structuring and research attention, e.g., before and after the intervention via diagnostic interviews, could have led to the overestimation of effects compared with interventions in routine care due to the Hawthorne effect [83]. Additionally, participants were characterized by high symptom severity at baseline assessment giving them more room for improvement compared to samples with subclinical symptoms. Adherence and motivation were increased by automated reminders and telephone reminders for questionnaire completion. Fourth, our findings can be generalized to similar settings. Thus, our results may be applicable to university students in Western countries with similar study characteristics. Finally, in health economic evaluations [84], a standard care comparator (e.g., face-to-face CBT) is recommended rather than a waitlist control group to avoid potential nocebo effects [85].

### 7.4. Clinical Implications and Future Research

The study results support the idea that IMIs could be a low-threshold, effective, and affordable way to reduce the adverse effects of social anxiety disorder. The advantage of an IMI is that compared with standard therapies, the marginal cost decreases when extending coverage or increasing the uptake of the intervention [86]. Low marginal costs and the scalability of IMIs enable them to be used as public health tools to generate effects at the population level compared with guided or face-to-face treatments that are not as scalable.

Moreover, based on the nature of social anxiety disorder, patients tend to avoid face-to-face contact with the therapist for fear being negatively evaluated. IMIs offer a low-threshold approach to help those affected without using face-to-face (F2F) treatments. This preference may also increase the use of unguided approaches compared with standard therapies. Therefore, future research should evaluate head-to-head comparisons of self-help IMIs with guided IMIs and F2F treatments.

Furthermore, in our study, only students who met the diagnostic criteria were included. Nevertheless, the students who were interested in participating but showing subclinical symptoms of SAD increased threefold. Thus, further research is needed to investigate the preventive effect of this IMI in students at risk of developing SAD. The application of this IMI across the range of mild to severe symptoms of SAD may better fit the requirements of a student mental health service at a university. However, under uncontrolled naturalistic conditions, the lack of research attention and reminders may decrease the effectiveness of unguided IMIs. Implementation studies could further examine the uptake and effectiveness of unguided IMIs under routine care conditions.

The German SAD treatment guidelines [87] recommend IMIs based on cognitive-behavioral therapy to bridge the waiting times or accompanying face-to-face treatment. Moreover, the results of our study add to the emerging evidence base in support of recommending IMIs as a viable treatment option in clinical guidelines. An official recognition of IMIs as a treatment option for SAD would help bridge the current treatment gap. During the COVID-19 pandemic, the barriers of treatment utilization increased and SAD symptoms in students were maintained due to minimal social contact and isolation [88]. Untreated persons generate long-term costs caused by persistent social anxiety symptoms, such as low academic performance, subsequent study dropouts, and worse job prospects, which are not included in our health economic evaluation. For the future, larger studies with longer follow-up periods are needed to investigate the full extent of SAD from a cost viewpoint.

## 8. Conclusion

This study strengthens the existing evidence confirming that internet-based self-help interventions for SAD can generate and sustain a significant and favorable effect in reducing social anxiety symptoms in a cost-effective way. Given the positive effects of the intervention, the implementation of this IMI as part of a student's healthcare management at the university would be essential.

## Figures and Tables

**Table 1:** Results of the ANCOVAs and Cohen's *d* for the primary and secondary outcome measures (ITT sample) at 6-month follow-up (T3).

Outcome	T3 between-group effect			T3 within-group effect	T3 within-group effect
	*d* (95%)	*F*_1,197_	*p*	IG	WLC
Primary outcome					
SPS	0.83 (0.54; 1.1)	55.01	<.001	1.27 (0.96; 1.57)	0.38 (0.10; 0.66)
SIAS	0.59 (0.30; 0.87)	49.03	<.001	1.30 (0.99; 1.60)	0.39 (0.11; 0.67)
Secondary Outcome					
BDI-II	0.45 (0.17; 0.73)	7.21	<.001	0.59 (0.31; 0.87)	0.17 (-0.11; 0.44)
BSI	0.38 (0.10; 0.66)	7.41	<.001	0.52 (0.24; 0.81)	0.17 (0.11; 0.45)
LSAS	0.76 (0.47; 1.05)	54.92	<.001	1.30 (1.00; 1.61)	0.37 (0.09; 0.65)
IIP-64	0.60 (0.32; 0.89)	44.92	<.001	1.16 (0.86; 1.46)	0.32 (0.04; 0.60)
FPES	0.54 (0.26; 0.82)	40.22	<.001	0.94 (0.65; 1.23)	-0.01 (-0.27; 0.29)
DPSOS-self	0.56 (0.82; 0.55)	21.43	<.001	0.68 (0.40; 0.97)	-0.06 (-0.34; 0.22)
DPSOS-others	0.58 (0.30; 0.86)	31.27	<.001	0.80 (0.51; 1.09)	-0.01 (-0.29; 0.27)
AQoL	0.23 (0.50; 0.05)	3.92	<0.01	0.66 (0.38; 0.95)	0.32 (0.04; 0.59)
EQ-5D	-0.08 (-0.36; 0.20)	2.14	>0.05	0.14 (-0.14; 0.42)	0.15 (-0.13; 0.42)

The analysis of covariance with baseline levels as covariates (at T0) was used. ANCOVA: analysis of covariance; IG: intervention group; ITT: intention-to-treat; M: means; SD: standard deviation; WLC: waitlist control group.

**Table 2:** Means and standard deviations for the IG and the WLC group (ITT sample).

Outcome	T0				T1^a^				T2^a^			
	IG		WLC		IG		WLC		IG		WLC	
	M	SD	M	SD	M	SD	M	SD	M	SD	M	SD
Primary outcome												
SPS	34.36	11.79	35.71	13.54	21.03	11.54	30.63	13.72	19.48	11.61	30.32	14.38
SIAS	51.47	11.23	48.71	12.92	36.72	13.86	44.36	14.05	35.10	13.78	43.40	14.39
Secondary outcome												
BDI-II	12.68	8.23	12.97	7.71	8.12	6.71	11.88	8.16	8.43	5.94	11.65	8.17
BSI	0.86	0.49	0.92	0.56	0.56	0.40	0.81	0.57	0.62	0.42	0.82	0.60
LSAS	77.61	16.87	76.96	19.57	58.82	20.45	72.51	22.17	52.08	21.87	69.10	22.64
IIP-64	1.71	0.39	1.66	0.43	1.34	0.47	1.5	0.48	1.17	0.53	1.5	0.56
FPES	43.82	11.00	39.90	13.00	36.17	13.49	39.95	14.6	33.19	11.54	40.06	13.62
DPSOS-self	16.76	4.91	15.85	5.68	14.35	5.42	16.06	5.89	13.47	4.69	16.16	4.89
DPSOS-others	42.51	11.93	40.16	12.56	36.11	14.81	40.60	14.81	32.27	13.59	40.31	14.03
AQoL	0.57	0.14	0.58	0.17	0.68	0.16	0.61	0.18	0.67	0.16	0.63	0.19
EQ5D	0.94	0.08	0.92	0.11	0.95	0.08	0.93	0.11	0.93	0.09	0.93	0.09

^a^Missing data imputed by multiple imputation. IG: intervention group; ITT: intention-to-treat; M: means; SD: standard deviation; WLC: waitlist control group.

**Table 3:** Average costs per participant (in €) by study arm at 6-month follow-up.

	IG (*n* = 100)		WLC (*n* = 100)		Incremental costs
	Mean (SD) (€)		Mean (SD) (€)		Difference (€)
Intervention	178.50		—	—	**+178.50**
Healthcare costs					
Physician services	18.90	(39.59)	38.06	(104.37)	-19.16
Mental Healthcare	114.31	(294.02)	84.81	(215)	+29.50
Inpatient care	0	(0)	21.97	(154.57)	-21.97^a^
Day care	0	(0)	47.42	(474.25)	-47.42
Nonphysician services	12.58	(36.61)	24.93	(136.43)	-12.35
Prescription drugs	21.70	(77.65)	23.11	(114.84)	-1.40
	167.49	(333.33)	240.31	(679.24)	-72.82

Patient and family costs					
Over-the-counter drugs	19.52	(36.08)	30.36	(64.13)	-10.83
Opportunity costs	61.78	(131.52)	130.27	(364.33)	-68.49
Travel expenses	8.67	(22.84)	19.10	(84.96)	-10.42
Domestic help/informal care	22.97	(92.16)	23.46	(131.93)	-0.49
	112.95	(220.88)	203.19	(475.86)	-90.24

Productivity losses					
Absenteeism (work)	219.48	(405.15)	203.13	(568.15)	+16.35
Presenteeism (work)	172.06	(331.87)	415.30	(1013.47)	-243.24
	391.54	(676.38)	618.43	(1466.64)	-226.89
Total healthcare costs	345.99	(333.33)	240.31	(679.24)	+105.69
Total societal costs	850.49	(943.42)	1061.94	(1969.21))	-211.45^b^

Sensitivity analyses					
Absenteeism (studies)	500.69	(591.80)	723.52	(1057.56)	-222.83
Presenteeism (studies)	320.84	(354.25)	348.88	(476.02)	-28.04
	821.53	(789.47)	1072.40	(1370.93)	-250.87

Average costs per participant are based on the area-under-the-curve approach and an intention-to-treat sample (*N* = 200). ^a^Costs included one outlier that was handled using winsorization. ^b^Due to rounding, numbers may not add exactly to the totals provided. IG: intervention group; WLC: waitlist control group.

**Table 4:** Results of the main and sensitivity analysis based on 5000 bootstrap simulations.

	Outcome	Incremental costs (€) (95% CI)	Incremental effects (points) (95% CI)	ICER (€/points) (95% CI)	Distribution over the CE plane (%)				WTP (p)
					NE^b^	SE^c^	SW^d^	NW^e^	
Main analysis									
Societal perspective	Symptom-free status SPS (range: 0-1)	-321 (-862 to 66)	0.26 (0.15 to 0.37)⁣^∗∗^	Dominant (to 414)	8	92	—	—	0 (0.92); 500 (0.98); 1000 (1)
	Symptom-free status SIAS (range: 0-1)	-324 (-774 to 125)	0.24 (0.14 to 0.34)⁣^∗∗^	Dominant(to 415)	8	92	—	—	0 (0.92); 500 (0.98); 1000 (1)
	AQoL QALYs (range: 0-1)	-319 (-831 to 64)	0.046⁣^∗∗^ (0.024 to 0.68)	Dominant (to 2447)	7	93	—	—	0 (0.93); 1000 (0.95); 2000 (0.97); 3000 (0.98); 10,000 (1)
Healthcare perspective	Symptom-free status SPS (range: 0-1)	81 (-105 to 200)	0.255 (0.144 to 0.366)⁣^∗∗^	348 (-284 to 1069)	86	14	—	—	0 (0.14); 500 (0.70); 1000 (0.97); 2000 (1)
	Symptom-free status SIAS (range: 0-1)	79 (-109 to 198)	0.244 (0.14 to 0.34)⁣^∗∗^	348 (-304 to 1043)	86	14	—	—	0 (0.14); 500 (0.69); 1000 (0.97); 2000 (1)
	AQoL QALYs (range: 0-1)	81 (-105 to 200)	0.046 (0.024 to 0.68)⁣^∗∗^	1945(-1521 to 6631)	86	14	—	—	0 (0.14); 1000 (0.32); 2000 (0.54); 3000 (0.73); 6000 (0.96)

Sensitivity analyses							—	—	
Diagnostic status									
Societal	Diagnostic status (range: 0-1)	-323 (-860 to 64)	0.3 (0.16 to 0.43)⁣^∗∗^	Dominant (to 332)	8	92	—	—	0 (0.92); 500 (0.99); 1000 (1)
Healthcare	Diagnostic status (range: 0-1)	79 (-107 to 198)	0.3 (0.16 to 0.43)⁣^∗∗^	288 (-254 to 900)	14	86			0 (0.14); 500 (0.79); 1000 (0.98)
EQ-5D									
Societal	QALYs (range: 0-1)	-319 (-829 to 63)	-0.00049 (-0.0166 to 0.0174)	112,106^a^	5	48	45	2	0 (0.99); 10,000 (0.89); 100,000 (0.60)
Healthcare	QALYs (range: 0-1)	80 (-100 to 210)	-0.00059 (-0.016 to 0.015)	Non-dominant	39	9	7	45	0 (0.15); 10,000 (0.26); 100,000 (0.48)
Increased intervention costs									
+50% intervention costs	QALYs (range: 0-1) (societal)	-230 (-741 to 153)	0.046 (0.024 to 0.68)	Dominant (to 4859)	15	85	—	—	0 (0.84); 1000 (0.89); 2000 (0.92); 3000 (0.95); 10,000 (1)
	QALYs (range: 0-1) (healthcare)	170 (-15 to 289)	0.046 (0.024 to 0.68)⁣^∗∗^	3995 (325 to 9878)	98	2	—	—	0 (0.02); 1000 (0.06); 2000 (0.16); 3000 (0.34); 10,000 (0.98)
+100% intervention costs	QALYs (range: 0-1) (societal)	-140 (-651 to 242)	0.046 (0.024 to 0.68)	Dominant (to 7417)	28	72	—	—	0 (0.72); 1000 (0.79); 2000 (0.84); 3000 (0.89); 10,000 (0.99)
	QALYs (range: 0-1) (healthcare)	259(113 to 404)	0.046 (0.024 to 0.67)⁣^∗∗^	6045 (2001 to 13,346)	100	—	—	—	0 (0.00); 1000 (0.0); 2000 (0.02); 3000 (0.9); 10,000 (0.92)

Costs are expressed in Euros (reference year: 2017). The SUREG model included significant outcome predictors (predictors for costs were age and treatment experience; predictors for outcome effects were baseline variables for each outcome). ^a^The dependably accurate 95% confidence interval for this distribution cannot be defined because there is no line through the origin that excludes alpha/2 of the distribution. ^b^The northeast quadrant of the CE plane, indicating that intervention is more effective and more costly. ^c^The southeast quadrant of the CE plane, indicating that intervention is more effective and less costly. ^d^The northwest quadrant of the CE plane, indicating that intervention is less effective and more costly. ^e^The southwest quadrant of the CE plane, indicating that intervention is less effective and less costly. ⁣^∗∗^*p* < 0.05. CI: confidence interval; ICER: incremental cost-effectiveness ratio; WTP: willingness to pay.

## Data Availability

The datasets generated and/or analyzed during the current study are available from the corresponding author on reasonable request.

## Additional Points

*Key Points*. (i) Unguided internet- and mobile-based interventions can induce and maintain favorable effects in students with social anxiety disorder over a period of 6 months. (ii) The mobile interventions have an acceptable likelihood of cost-effectiveness. (iii) It would be helpful to integrate such mobile interventions into routine mental healthcare at universities.

## Supplementary Materials

Supplementary Materials Table S1: unit costs for the type of health service utilized by the participants. Table S2: baseline sample characteristics. Table S3: treatment response, symptom-free status, and symptom deterioration at 6-month follow-up.
