# Supplementary material for: Long-Term Outcomes and Cost-Effectiveness of an Internet-Based Self-Help Intervention for Social Anxiety Disorder in University Students: Results of a Randomized Controlled Trial
Source: Depress Anxiety. 2023 Nov 17;2023:7912017. doi: 10.1155/2023/7912017 (PMC11921830; doi:10.1155/2023/7912017)
Supplement: Supplementary Materials — Table S1: unit costs for the type of health service utilized by the participants. Table S2: baseline sample characteristics. Table S3: treatment response, symptom-free status, and symptom deterioration at 6-month follow-up. [file 7912017.f1.pdf]

# Supplementary material

**Table S1** Unit costs for the type of health service utilized by the participants

| Health service type | Unit | Costs (€)[a] |
|---|---|---|
| Physician | Contact | 21.53 |
| Orthopedist | | 26.71 |
| Specialists for internal medicine | | 66.47 |
| Dermatologist | | 20.26 |
| Urologist | | 26.07 |
| ENT specialist | | 29.09 |
| Neurologist | | 48.64 |
| Psychotherapist | | 82.17 |
| Logopedics / speech therapy | Contact | 40.56 |
| Physiotherapy | | 17.30 |
| Ergotherapy / occupational therapy | | 39.01 |
| Mean remedies | | 32.29 |
| General hospital / inpatient | Day | 648.11 |
| Mental hospital / inpatient | | 348.26 |
| General hospital / day patient | | 421.27 |
| Mental hospital / day patient | | 226.37 |
| Rehabilitation /outpatient | Day | 49.43 |
| Rehabilitation /day patient | | 93.81 |
| Rehabilitation /inpatient | | 138.19 |
| University counselling center | Contact | 48.40 |
| **Patient and family costs** | **Unit** | **Costs (€)** |
| Costs for travel[b] | Per kilometer | €0.30 (US$0.40) |
| Opportunity costs[c] | Per hour | €22.85 (US$30.30) |
| Informal Care from friends and family[d] | Per hour | €19.63 (US$25.67) |

[a] Unit costs were calculated or adjusted by the German consumer price index[89] for the year 2017
[b] Schmidt, L. Einkommenssteuergesetzbuch (EStG) [German Income Tax Code] (2017)
[C] Bock, J.-O. et al. Standardisierte Bewertungssätze aus gesellschaftlicher Perspektive für die gesundheitsökonomische Evaluation. 55 (Nomos Verlagsgesellschaft mbH & Co. KG, 2015)

[d] Bock, J.-O. et al. Ermittlung standardisierter Bewertungssätze aus gesellschaftlicher Perspektive für die gesundheitsökonomische Evaluation. [Calculation of Standardised Unit Costs from a Societal Perspective for Health Economic Evaluation] Das Gesundheitswes. 77, 53–61 (2014)

**Table S2** Baseline sample characteristics

| Characteristics | All participants (*N* = 200) | IG (*n* = 100) | WLC (*n* = 100) |
|---|---|---|---|
| | N (%) | N (%) | N (%) |
| **Sociodemographic characteristics** | | | |
| Age (M, SD) | 26.70 (6.34) | 26.71 (6.08) | 26.68 (6.61) |
| Sex, female | 124 (62%) | 63 (63%) | 61 (61%) |
| Married or in a relationship | 102 (51.0%) | 52 (52.0%) | 50 (50.0%) |
| **Citizenship** | | | |
| Germany | 150 (75%) | 74 (74.0%) | 76 (76.0%) |
| Switzerland | 40 (20%) | 21 (21.0%) | 19 (19.0%) |
| EEA member | 3 (1.5%) | 1 (1.0%) | 2 (2.0%) |
| No EEA member | 7 (3.5 %) | 4 (4.0%) | 3 (3.0%) |
| **Study characteristics** | | | |
| Full-time student | 170 (85.0%) | 86 (86.0%) | 84 (84.0%) |
| Part-time student | 30 (15.0%) | 14 (14.0%) | 16 (16.0%) |
| Semester (M, SD) | 5.09 (3.38) | 4.65 (3.15) | 5.53 (3.56) |
| **Work characteristics** | | | |
| Employed | 106 (53.0%) | 58 (58.0%) | 48 (48.0%) |
| Full-time employed | 17 (8.5%) | 9 (9.0%) | 8 (8.0%) |
| **Chronic conditions** | | | |
| Any chronic condition | 127 (64%) | 66 (66%) | 61 (61%) |
| **Treatment utilization** | | | |
| Previous psychotherapy | 68 (34.0%) | 38 (38.0%) | 30 (30.0%) |
| Medication at T0 | 5 (2.5%) | 3 (3.0%) | 2 (2.0%) |

*EEA* European Economic Area, *M* mean, *SD* standard deviation

**Table S3** Treatment response, symptom-free status, and symptom deterioration at 6-month follow-up

| | T2[a] | | | | |
|---|---|---|---|---|---|
| | IG (n=100) | WLC (n=100) | $\chi 2(1)$ | *p* | NNT (95% CI) |
| Outcome | N (%) | N (%) | | | |
| **Reliable improvement** | | | | | |
| SPS | 77 | 40 | 28.20 | < .001 | 2.7 (2.0; 4.1) |
| SIAS | 72 | 28 | 38.72 | < .001 | 2.3 (1.8; 3.2) |
| **Symptom-free status** | | | | | |
| SPS | 49 | 21 | 17.23 | < .001 | 3.6 (2.5; 6.5) |
| SIAS | 32 | 11 | 13.06 | < .001 | 4.8 (3.1; 10.0) |
| **Symptom deterioration** | | | | | |
| SPS | 3 | 26 | 21.33 | < .001 | - |
| SIAS | 5 | 29 | 20.41 | < .001 | - |

[a] Missing data imputed by multiple imputation

*CI* confidence interval, $\chi 2$ chi square test, *IG* intervention group, *NNT* Number Needed to Treat, *WLC* waitlist control group
